# Trauma to the Temporomandibular Joint Following Tooth Extraction via Dental Students

**DOI:** 10.5812/kowsar.22517464.3432

**Published:** 2012-01-15

**Authors:** Mohammad Ali Dolatabadi, Esshagh Lassemi

**Affiliations:** 1 Department of Oral and Maxillofacial Surgery, Azad University of Medical Sciences, Dental Branch, Tehran, IR Iran

**Keywords:** Temporomandibular Joint Disorders, Tooth Extraction

*Dear Editor,*

There is an important point related to temporomandibular joint (TMJ) injuries and derangement due to trauma from dental extractions by dental students. This subject should be an utmost cause for concern for dentists and dental practitioners. Because if not treated it may become chronic. This issue has been noted by many researchers in academic dental training centers worldwide. The results of many studies in this field have shown surprisingly high prevalence rates of TMJ injuries (50-63 %) after extracting mandibular teeth by dental students (1). About 60 % of patients develop pain, clicking and limitation of mouth opening after extraction of molar teeth (2, 3). At the Azad University Dental School (Oral and Maxillofacial Surgery Department) a similar study was done. The result was similar to those of other centers. Excessive uncontrolled force used to extract mandibular molars is one of the major factors predisposing to the development of temporomandibular disorders (TMD). Therefore, continued research in this field is warranted to increase awareness of this issue and to implement procedures to prevent TMD such as shortening procedure time for the patient, less than maximum mouth opening of the patient undergoing tooth extraction, use of controlled force by the student when extracting as well as providing manual support of the mandible upon extraction of mandibular teeth especially molars.
